# Antibiotics in childhood pneumonia: how long is long enough?

**DOI:** 10.1186/s41479-016-0006-x

**Published:** 2016-05-11

**Authors:** Keith Grimwood, Siew M. Fong, Mong H. Ooi, Anna M. Nathan, Anne B. Chang

**Affiliations:** 1 Menzies Health Institute Queensland, Griffith University and Gold Coast Health, Gold Coast, Queensland 4222 Australia; 2 Division of Pediatric Infectious Diseases, Pediatric Department, Hospital Likas, Kota Kinabalu, Sabah Malaysia; 3 Department of Pediatrics, Sarawak General Hospital, Sarawak, Malaysia and Institute of Health and Community Medicine, Universiti Malaysia Sarawak, Sarawak, Malaysia; 4 Department of Paediatrics, University of Malaya, Kuala Lumpur, Malaysia; 5 Menzies School of Health Research, Charles Darwin University, Darwin, Northern Territory Australia; 6 Department of Respiratory and Sleep Medicine, Lady Cilento Hospital and Institute of Health and Biomedical Innovation, Queensland University of Technology, Brisbane, Queensland Australia

**Keywords:** Pneumonia, Antibiotics, Child

## Abstract

Improved access to healthcare, vaccines and treatment with antibiotics has reduced global mortality from childhood community-acquired pneumonia. However, as respiratory viruses are responsible for most episodes of pneumonia, important questions remain over who should receive these agents and the length of each treatment course. Worldwide concerns with increasing antibiotic resistance in respiratory pathogens and appeals for more prudent antibiotic prescribing provide further urgency to these clinical questions. Unfortunately, guidelines for treatment duration in particular are based upon limited (and often weak) evidence, resulting in national and international guidelines recommending treatment courses for uncomplicated pneumonia ranging from 3 to 10 days. The advantages of short-course therapy include a lower risk of developing antibiotic resistance, improved adherence, fewer adverse drug effects, and reduced costs. The risks include treatment failure, leading to increased short- or long-term morbidity, or even death. The initial challenge is how to distinguish between bacterial and non-bacterial causes of pneumonia and then to undertake adequately powered randomised-controlled trials of varying antibiotic treatment durations in children who are most likely to have bacterial pneumonia. Meanwhile, healthcare workers should recognise the limitations of current pneumonia treatment guidelines and remember that antibiotic course duration is also determined by the child’s response to therapy.

Community-acquired pneumonia is the leading global cause of childhood morbidity and mortality. Annually, there are an estimated 120–160 million clinical pneumonia episodes worldwide, causing 14 million hospitalisations and almost one million deaths in children aged <5 years [1, 2]. Although respiratory viruses are the most common pathogens associated with childhood pneumonia, most deaths are attributed to *Streptococcus pneumoniae* and *Haemophilus influenzae* type b [3]. Consequently, antibiotics have reduced pneumonia-related morbidity and mortality. Nevertheless, several knowledge gaps exist with prescribing antibiotics for pneumonia, including the optimal length of treatment required. These limitations are evident in both national and international guidelines, which have had to rely upon expert opinion and weak levels of evidence from a small number of clinical trials with substantial methodological limitations [4–7]. A good example of these difficulties is the range of recommendations provided on treatment duration for uncomplicated childhood pneumonia [5, 6]. This raises several questions for healthcare workers when determining how long they should be giving antibiotics to a child with pneumonia.

## What factors influence decisions on antibiotic duration?

Several factors are considered when both choosing an antibiotic to treat a suspected case of bacterial pneumonia and determining how long it should be given. These include: (i) clinical presentation and severity; (ii) assumed bacterial aetiology based upon the child’s age, vaccination status, underlying co-morbidities and the local pathogen antibiotic susceptibility profiles; and (iii) cost, availability, tolerability, and ease of administration (e.g. frequency and palatability) of the chosen agent that may influence treatment adherence.

In clinical practice, the optimal duration of antibiotic treatment depends upon whether the pneumonia is straightforward or complicated (e.g. empyema or systemic infection involving other organs); if underlying medical disorders are present (e.g. malnutrition, human immunodeficiency virus infection, or chronic cardiopulmonary disease); the nature of the causative pathogen, adequacy of source control, and the patient’s response to treatment.

In uncomplicated pneumonia the advantages of a short-treatment course include a lower risk of developing antibiotic resistance, improved adherence, fewer adverse effects, and decreased costs [6, 8]. The main danger though of shortened therapy in young children is treatment failure from delayed or incomplete eradication of the infecting pathogen(s), risking additional morbidity and injury to the developing lungs and possibly a greater chance of impaired lung function, chronic obstructive pulmonary disease and bronchiectasis later in life [9].

### It is really bacterial pneumonia?

Most studies on antibiotic duration were undertaken in low- and low-to-middle-income countries where the burden of pneumonia is greatest. Unfortunately, the diagnosis of bacterial pneumonia in these settings is also the most uncertain as it relies upon healthcare workers following clinical algorithms without adequate laboratory and radiographic support. Furthermore, no diagnostic gold standard for pneumonia exists, and there are major difficulties differentiating between viral and bacterial pneumonia clinically and radiographically, let alone obtaining an accurate microbiological cause [10].

### What are the current recommendations and what are their limitations?

As most childhood pneumonia deaths occur out of hospital in the low-resource settings of sub-Saharan Africa and Southern Asia, the diagnostic algorithms used by the World Health Organization (WHO) are designed to reduce mortality, sacrificing specificity for sensitivity [4]. Otherwise healthy children with suspected clinical pneumonia are managed as outpatients and receive either 3 days of high-dose oral amoxicillin (80–90 mg/kg/day) if tachypnoeic alone, or 5 days if subcostal recession is also present [4]. Those with severe clinical pneumonia accompanied by danger signs (e.g. dehydration, seizures, or altered consciousness) receive parenteral penicillin (or ampicillin) and gentamicin as first-line agents for at least 5 days. These recommendations are based upon several large randomised controlled trials (RCTs) of oral vs. parenteral antibiotics and 3 vs. 5 days of oral antibiotic treatment in children from developing countries [7, 11]. A recent systematic review published in the journal [7] found three RCTs from developing countries comparing short (3 days) vs. standard (5 days) oral antibiotic treatments in children with non-severe (tachypnoea alone) pneumonia. These studies were conducted in either India or Pakistan and each involved >2000 children aged 2–59 months [12–14]. Each reported that 3 days was either equivalent [12] or not statistically different [13, 14] to 5 days treatment. However, the validity of these three studies is questionable. The follow-up was limited to only 14 days and although failure rates ranged from 9.5 % to 21 %, just a single death occurred in a 3-month old infant amongst the 6197 trial subjects, a much lower case fatality than expected for pneumonia in these settings [2, 3]. Almost half the subjects were infants, as many as 22 % had wheeze, and pneumonia was diagnosed following the WHO clinical algorithm [4]. Only one study included chest radiographs [12], where just one in seven children with clinical pneumonia had abnormal radiographic findings. Consequently, these studies of treating non-severe pneumonia in developing countries are limited by inherent biases towards equivalence of varying treatment durations, since many (if not most) participating subjects were unlikely to benefit from antibiotics as they had bronchiolitis, viral pneumonia, or virus-associated wheeze. Indeed, a recent double-blind RCT in 900 children aged 2–59 months from Pakistan with WHO-diagnosed non-severe pneumonia found equivalent clinical outcomes in those receiving either 3 days of oral amoxicillin or placebo with cumulative treatment failure rates by day 5 of 13.5 % and 17.6 %, respectively, while once again no deaths were reported [15].

In contrast to developing countries, criteria for diagnosing childhood pneumonia in developed nations often require chest radiographic confirmation, especially for hospitalised cases [5]. Nevertheless, little information is available guiding treatment duration [6, 7], although a recent small, single-centre, three-arm RCT from Israel of 140 non-hospitalised children aged <5 years with likely bacterial pneumonia (based on clinical criteria, chest radiographic consolidation, and raised white blood cell counts) found that the 40 % failure rate of a 3 day course of amoxicillin was unacceptably high, while no failures were reported in those receiving either a 5 or 10 days course of the antibiotic [16]. These data help support current national guidelines from developed countries recommending at least 5 days of antibiotics for children suspected of bacterial pneumonia [5, 6].

### So, what is required?

The guidelines for the treatment duration of pneumonia are based upon limited and often weak evidence [4–7]. The situation is not helped by the important knowledge gaps that still remain regarding how to best identify children with pneumonia, including how to reliably differentiate between bacterial and non-bacterial causes [10]. Healthcare workers in resource poor settings in particular need access to validated, simple, and inexpensive point-of-care diagnostic tests. Unfortunately, none are likely to be available soon. While new technologies such as gene expression signatures show considerable promise for identifying aetiological pathogens in pneumonia, these and other molecular-based platforms are unlikely to be made available in the foreseeable future to low- and middle-income countries where the burden of pneumonia is greatest. Meanwhile, although there is mounting global concern over rising rates of antibiotic resistance resulting in increased calls for shorter treatment courses, it is important to remember that an ineffective short antibiotic treatment course for pneumonia is still the worst strategy when either it is not needed (e.g. for viral respiratory infections) or when it results in treatment failure, risking death, increased morbidity and/or long-term sequelae [8–10].

Clearly, more robust evidence for antibiotic treatment duration for pneumonia is needed. A good start would be to undertake additional RCTs in sub-Saharan Africa and Asia (where feasible sample sizes are possible), recruiting subjects with a greater probability of bacterial infection (based on clinical severity or radiographic criteria). Meanwhile, healthcare workers should recognise the limitations of current “one size fits all” pneumonia treatment guidelines and remember that the duration of antibiotic therapy is also determined by individual host and pathogen factors and how the child responds to treatment.
